# Exploring the Factors Associated with Mental Health Attitude in China: A Structural Topic Modeling Approach

**DOI:** 10.3390/ijerph191912579

**Published:** 2022-10-01

**Authors:** Ruheng Yin, Rui Tian, Jing Wu, Feng Gan

**Affiliations:** 1 School of Art, Culture and Tourism Industry Think Tank Chinese Art Evaluation Institute, Southeast University, Nanjing 211189, China; 2 School of Sociology and Population Studies, Nanjing University of Posts and Telecommunications, Nanjing 211189, China

**Keywords:** mental health attitude, COVID-19, China, social media, Weibo, structural topic modeling, text analysis

## Abstract

Mental health attitude has huge impacts on the improvement of mental health. In response to the ongoing damage the COVID-19 pandemic caused to the mental health of the Chinese people, this study aims to explore the factors associated with mental health attitude in China. To this end, we extract the key topics in mental health-related microblogs on Weibo, the Chinese equivalent of Twitter, using the structural topic modeling (STM) approach. An interaction term of sentiment polarity and time is put into the STM model to track the evolution of public sentiment towards the key topics over time. Through an in-depth analysis of 146,625 Weibo posts, this study captures 12 topics that are, in turn, classified into four factors as stigma (*n* = 54,559, 37.21%), mental health literacy (*n* = 32,199, 21.96%), public promotion (*n* = 30,747, 20.97%), and social support (*n* = 29,120, 19.86%). The results show that stigma is the primary factor inducing negative mental health attitudes in China as none of the topics related to this factor are considered positive. Mental health literacy, public promotion, and social support are the factors that could enhance positive attitudes towards mental health, since most of the topics related to these factors are identified as positive ones. The provision of tailored strategies for each of these factors could potentially improve the mental health attitudes of the Chinese people.

## 1. Introduction

Mental health is a major public health issue in China. According to the survey conducted by Huang et al., the 12-month prevalence rate of any mental disorder was 9.3% and the lifetime prevalence rate of any mental disorder was 16.6% [1]. The outbreak and rapid spread of the COVID-19 pandemic have severely worsened the mental health of the Chinese people due to the contagious nature of the virus [2], the daily updated case numbers [3], and quarantine and lockdown policies [4], leading to mental health symptoms such as stress disorder, depression, anxiety, and post-traumatic stress disorder (PTSD) [5,6]. The improvement of mental health in China is therefore of great urgency and importance, which arouses the interest of many researchers.

The prior literature mainly focused on the identification and treatment of mental health conditions. For example, Wang et al. examined the psychological problems suffered by the Chinese people during the COVID-19 pandemic and suggested using online trauma-focused psychotherapy as a countermeasure [7]. Shi et al. revealed a pronounced and prolonged mental health burden on the Chinese public caused by the COVID-19 pandemic, and pointed out that the mental health status of the vulnerable populations should be carefully monitored throughout the pandemic [8]. Hao et al. confirmed the negative psychological impact experienced by people during the COVID-19 pandemic and provide insights into the development of a new immunopsychiatry service [9].

However, mental health attitude in China has yet to be sufficiently investigated to fully understand where further improvement is needed. Mental health attitude represents the way of perceiving and treating people with mental health problems [10]. Positive mental health attitudes can effectively enhance mental health literacy, reduce stigma, and strengthen social support [11], which collectively protect and facilitate mental health [12]. For this reason, the objective of this study is to investigate the factors associated with the mental health attitudes of the Chinese people and hopefully be able to translate the findings into appropriate advice to efficiently improve the mental health attitude in China.

The rest of the paper is arranged in five sections. In Section 2, we provide an overview of several studies on related topics. Section 3 explains the research methodology applied in this study, where the basic process of data collection and processing and the STM model setup are described. Then, in Section 4, we present the main results of this study, followed by Section 5, where all of the results are thoroughly discussed. At the end of the paper is Section 6, where we end with the conclusion of our study.

## 2. Literature Review

People with mental health problems have long been described as dangerous and violent [13]. The stigma against mental health problems is so deeply rooted that such negative views are not only common in the general public, but are also held by mental health providers [14]. Negative mental health attitudes pose serious mental health challenges as they often catalyze discrimination against people with mental health problems [15], making them reluctant to seek mental health care due to the fear of stigma from others [16].

Researchers have tended to explore the factors associated with mental health attitude. Previous efforts were almost exclusively conducted using surveys. For example, Bradbury utilized a cross-sectional survey design to assess the influence of age and gender on mental health attitude [17]. Kotera et al. used questionnaire surveys to evaluate shame-based attitudes toward mental health problems among Japanese workers [18]. However, despite their contributions, previous studies had several methodological limitations. First, traditional surveys are very time consuming, and survey data are often biased due to increasingly low response rates (usually well below 10%) [19,20]. In addition, surveys are carried out by asking participants to answer questions predesigned by the researchers, which makes it difficult to monitor mental health attitude and its dynamic changes in real time [21].

One way in which this study seeks to address these research limitations in the current literature is to take advantage of the large-scale and real-time user-generated data on social media platforms [22]. Social media has increasingly become a popular platform for information seeking and sharing [23]. Content produced on social media can rapidly spread among the public, triggering cascading effects that can affect people’s perceptions and beliefs [24]. This study used Weibo as the data source. Although often referred to as the Chinese equivalent of Twitter, Weibo is more like a mixture of features of Twitter and Facebook [25]. Weibo had 582 million monthly active users and 252 million daily active users by the first quarter of 2022 [26], and, as such, it is recognized as a unique platform to explore the attitudes of the Chinese people due to the sheer number of users [27]. Weibo has been widely used as a data source for mental health studies. For example, Tian et al. used Weibo data to assess the depression issues in China [28]. Liu et al. collected data from Weibo to examine the longitudinal change in mental health among active social media users during the COVID-19 pandemic [5]. To our knowledge, Weibo has not been tapped to examine mental health attitude.

This study also seeks to detect the factors associated with mental health attitudes from the latent topics discussed in mental health-related Weibo posts. At present, the most commonly used topic modeling method is Latent Dirichlet allocation (LDA) [29]. The LDA model assumes that a document consists of a series of latent topics, where each topic contains a set of words that help define the theme of the topic. LDA has been frequently used in public health studies, such as examining public response to public health policies [30], public opinions on environmental health threats [31], analyzing the media coverage of public health issues [32], and many more. However, LDA is not suitable for this study, as it cannot measure the evolution of mental health attitude due to the inability to evaluate topics using document-level covariates [33]. Therefore, this study used structural topic modeling (STM) to process the data. STM is a more recent application of the topic modeling approach that can expand the ability of the LDA model to consider metadata associated with the text using document-level covariates. For example, He et al. implemented an interaction term of review time and review extremity into the STM model to explore the evolution trend of the (dis)satisfaction of drug consumers from web-based drug reviews [28]. Pirri et al. used the value of influencer score and network influencer score as the covariates to explore the nature of topics posted by users and organizations on Twitter during World Lupus Day [34]. To our knowledge, this study is the first attempt to use STM to investigate mental health attitude.

This study utilized STM, a cutting-edge computerized machine learning model, to process mental health-related microblogs on Weibo, aiming to explore the factors associated with mental health attitude in China. Our approach not only helps capture the key topics of the Weibo discussions on mental health, but also reflects their dynamic changes over time. Specifically, what was of the utmost concern of the Chinese people about mental health, how positive (or negative) did they feel about these concerns, and what factors associated with mental health attitude in China can be distilled from these concerns? The findings will help deepen our understanding of the mental health attitudes of the Chinese people and serve as a trustworthy reference for mental health stakeholders to improve mental health attitude in China.

## 3. Methodology

**Figure 1:**
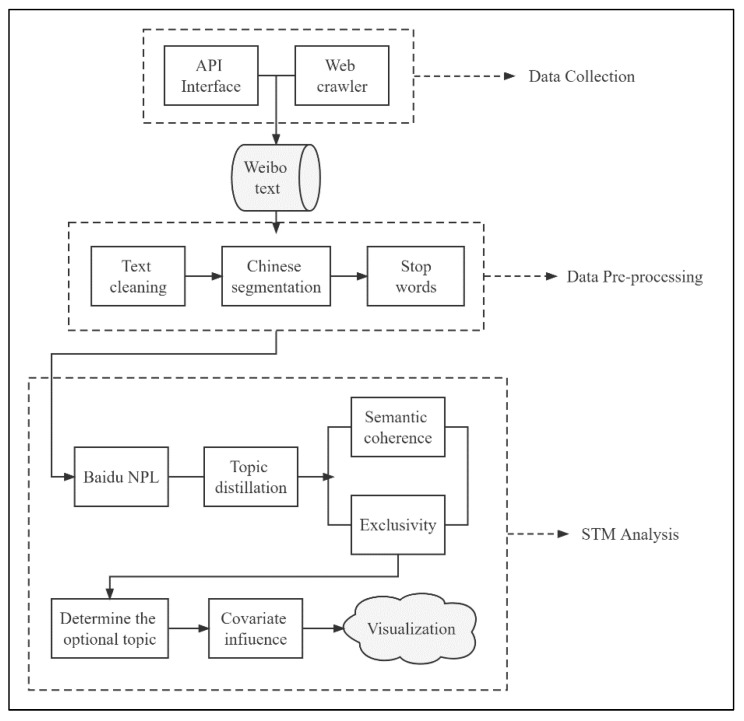
Framework of the data analysis process.

This study grows out of the conviction that conducting structural topic modeling analysis on the textual content extracted from Weibo posts offers us a vantage point to contribute to the understanding of the factors associated with mental health attitude in China. To achieve this research objective, this study proposes an effective research model to mine and examine data from Weibo (Figure 1).

### 3.1. Data Collection and Processing

Data collection began with identifying search terms. We extracted Weibo posts under several different search terms using Weibo public streaming application programming interface (API). With the help of an expert in the field of mental health, we checked the number and scope of the collected Weibo posts. Ultimately, Mental Health, Mental Problems, and Psychological Illness were identified as the search terms in this study. Weibo’s API allows us to query a maximum of 2000 posts, which resulted in insufficient data from the initial return. To address this issue, we wrote a web crawler with Python code to obtain data. By simulating logging into Weibo, the web crawler helps us circumvent the data-fetching restrictions. The information crawled included post time, post content, post location, poster gender, repost count, and follower count. Within the specified time frame (i.e., from August 2021 to July 2022), a total number of 174,749 Weibo posts were collected as sample data.

The second step is data cleaning. The nature of the textual content from Weibo posts is unstructured data. It often contains a variety of noise information such as URL, emoji, and punctuation, which could affect the accuracy of the text recognition. Therefore, sample data were filtered out using Pandas, a powerful and flexible open-source data manipulation tool in Python, to delete posts that were repeated, meaningless, or consisted of emoji only. In addition, we used prepDocument and plotRemoved to identify and remove low-frequency words. The final dataset included 146,625 Weibo posts.

The last step is data preprocessing. First, we conducted word segmentation on the sample posts using the Jieba package in Python. Word segmentation refers to splitting Chinese text (a sequence of Chinese characters) into individual words, which is a necessary step when processing Chinese text due to the lack of delimiters between words in written Chinese sentences. Jieba is one of the most effective Python Chinese word segmentation modules. Then, we removed stop words based on the user-defined stop word list.

### 3.2. STM Model Setup

Our primary research objective is to identify the factors associated with the mental health attitudes of the Chinese people. Of particular interest is to analyze their dynamic changes over time. To this end, this study selected sentiment polarity and time as the two covariates to build the STM model. We used the open-source NLP technique from Baidu to automatically label the sentiment polarity of the sample posts [35]. This NLP technique divides Weibo posts into positive and negative categories. Each post was tagged with a numerical sentiment value from 0 to 1, with 0 indicating a strongly negative emotion and 1 indicating a strongly positive emotion.

Another step in the STM that needs to be elucidated is the choice of the number of topics (k). The optimal number of topics is usually set to achieve the most substantive interpretation of the outcomes rather than the maximization of the topics [36]. This study used semantic coherence and exclusivity to determine the optimal number of topics. The semantic coherence metric measures the most probable words in a specified topic that occur together [37]. When this simultaneous occurrence is frequent, semantic coherence will thus reach its maximization. The exclusivity metric indicates that the most probable words will not appear in any other topics rather than the specified topic at the same time [38]. An analysis of semantic coherence and exclusivity conducted for the number of topics ranging from 10 to 20 revealed 12 as a satisfactory number of topics to explore in the study. Each topic consists of several terms with the highest (beta) β probabilities that represented the topic content (Y).

**Figure 2:**
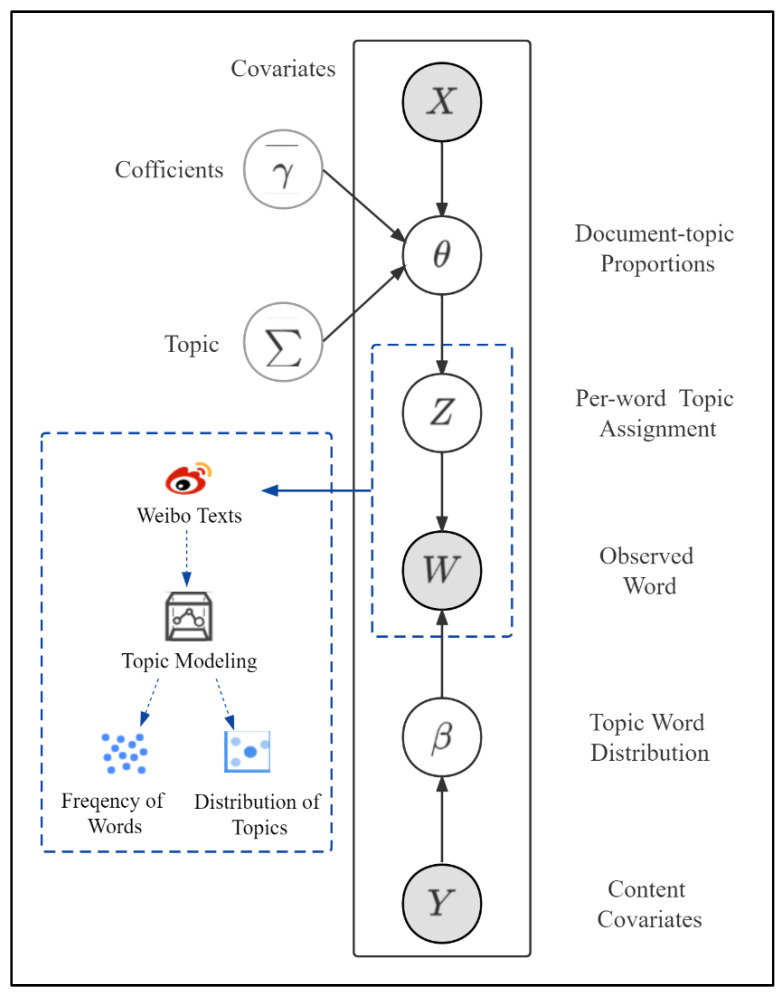
Plate diagram of structural topic model (STM).

This study used the STM package in R to conduct the topic modeling analysis. We used one-hot encoding to embed words into the STM model. One-hot encoding is an effective word embedding tool implanted in the STM package. After the identification of the topics, we estimated the impacts that sentiment polarity and time as covariates (X) had on topic prevalence, explored changes in public attitudes towards mental health over time, and aimed to provide timely and efficient health communication advice. The STM model is parameterized by document-specific covariates X and Y. Figure 2 represents the model diagram of the STM in this study.

## 4. Results

### 4.1. General Description

**Figure 3:**
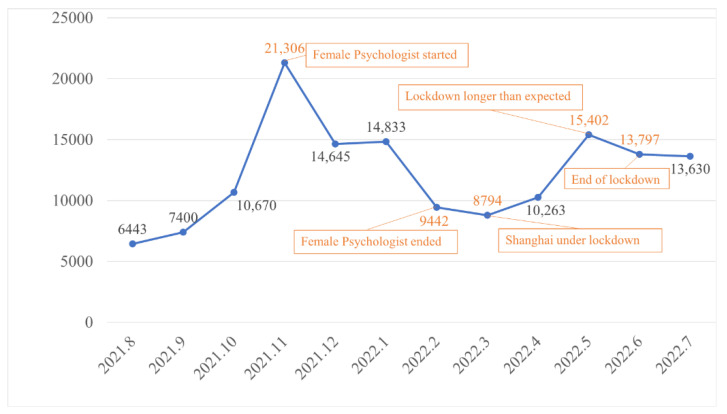
Total number of Weibo posts for 12 months from August 2021 to June 2022.

From the dataset composed of 146,625 Weibo posts, the overall Weibo activity on mental health during the period of the study is presented in Figure 3. In general, the number of mental health-related Weibo posts fluctuated over the months, with two obvious increases in November 2021 and May 2022, respectively.

The first increase occurred in the same month when the Chinese television show Female Psychologistpremiered on Youku, one of China’s top online video and streaming service platforms [39]. Female Psychologist is one of the few Chinese television shows that focus on mental health. It carefully picked seven mental health cases and offered an in-depth analysis of their causes. In the course of the show, some of the highly prevalent mental health problems in Chinese society were introduced to the Chinese audience, leading to extensive discussions on China’s social media platforms. The number of mental health-related Weibo posts reached over 20,000 in November 2021, representing a two-fold leap over the previous month. In addition, when the show ended in February 2022, the number of mental health-related Weibo posts dropped considerably.

The second increase happened in May 2022, which coincided with the 2022 Shanghai COVID-19 outbreak. The metropolitan city was shut down from March 2022 [40]. The lockdown lasted longer than expected, which incurred severe psychological crisis [41]. Two months into the complete lockdown in Shanghai, over 15,000 mental health-related Weibo posts were captured in May 2022. The end of the 75-day lockdown in Shanghai, which was on 1 June 2022, did not reduce the general public’s attention on mental health. Nearly 14,000 mental health-related Weibo posts were collected in June 2022.

### 4.2. Topic Summary

Table 1 shows the topics generated from the STM model based on the mental health-related Weibo posts we extracted from August 2021 to July 2022. The first column demonstrates the topic number. The second column represents the topic labels. It is worth noting that the topic labels are not generated automatically in the STM [42]. It is up to the researchers to assign the label of the topic with the help of the high-probability terms linked to each topic [43]. In this study, a specified label was determined for each topic using the authors’ unanimous judgments through an open discussion with one expert in the field of mental health on one hand, and one expert in the field of public health communication on the other. The third column shows the proportion of each topic. The fourth column lists the high-probability terms.

To better understand the meaning of the topics, we also examined the most representative Weibo posts of each clustered topic. Table 2 shows the topics and the associated Weibo posts.

### 4.3. Topic Sentiment Polarity Identification

**Figure 4:**
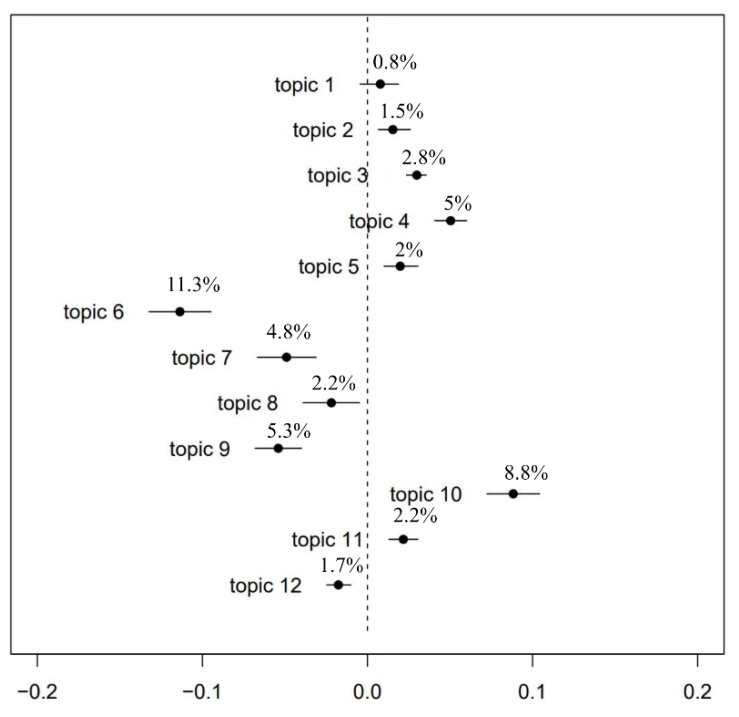
Topic prevalence based on sentiment polarity (positive vs. negative).

As latent topics in the Weibo discussions were captured in the STM model, the general public’s sentiment towards those topics also emerged. Figure 4 demonstrates the sentiment values of each topic. A topic will be identified as positive if the proportion of this topic in the positive Weibo posts is noticeably larger than in the negative ones, and vice versa [44]. The dotted line represents neutral. The points represent the mean values of the estimated differences, and the bars represent the 95% confidence intervals of the differences [33]. Take patient stories (topic 9) as an example: the proportion of negative Weibo posts of this topic is higher than that of negative reviews by 5.3%, and the difference exists at a 3.7–6.7% confidence interval. Therefore, this topic is identified as a negativetopic.

As shown in Figure 4, information seeking (topic 1), advice and sharing (topic 2), media promotion (topic 3), celebrity effect (topic 4), community effect (topic 5), encouragement (topic 10), and World Mental Health Day (topic 11) were identified as positive topics. Encouragement (topic 10) and community effect (topic 5) were both perceived as positive topics since the microblogs clustered in these two topics were mostly posted to motivate people with mental health problems. Mass media is extremely effective in promoting public health messages [45], which is reflected in the positive perceptions of media promotion (topic 3), celebrity effect (topic 4), and World Mental Health Day (topic 11) on Weibo. The positive sentiment towards information seeking (topic 1) and advice and sharing (topic 2) shows that knowledge about mental health could lead to the understanding of and support for mental health problems.

Public stigma (topic 6), self-stigma (topic 7), mental health service (topic 8), patient stories (topic 9), and symptoms description (topic 12) were considered negative topics. It is obvious that stigma is the major source of negative mental health attitudes in China since public stigma (topic 6), self-stigma (topic 7), and patient stories (topic 9) are all related to the stigmatization of people with mental health problems. This is in line with the common notion that people with mental illness should be feared and isolated [46]. The negative sentiment towards symptoms description (topic 12) shows that inadequate mental health literacy could encourage misperceptions of mental health problems, leading to negative mental health attitudes among the general public. Mental health service (topic 8) was also identified as a negative topic, signifying dissatisfaction with the quality and scale of mental health services in China.

### 4.4. Topic Sentiment Polarity Variations

**Figure 5:**
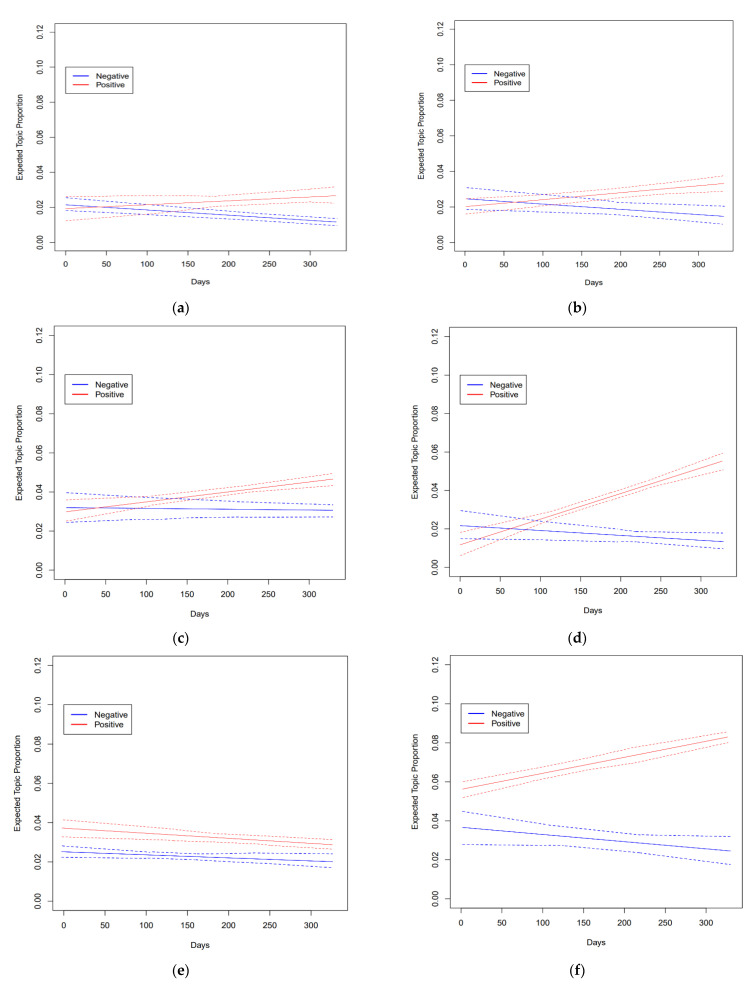

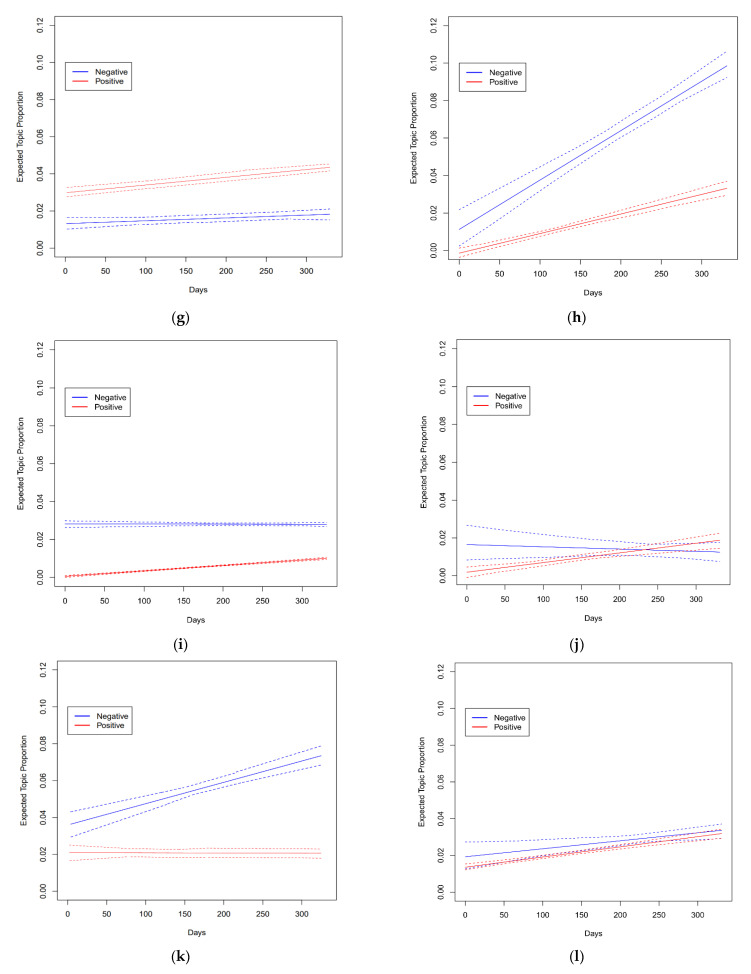
Change in topic prevalence based on sentiment polarity over time. (**a**) Information seeking (topic 1); (**b**) advice and sharing (topic 2); (**c**) media promotion (topic 3); (**d**) celebrity effect (topic 4); (**e**) community effect (topic 5); (**f**) encouragement (topic 10); (**g**) World Mental Health Day (topic 11); (**h**) public stigma (topic 6); (**i**) self-stigma (topic 7); (**j**) mental health service (topic 8); (**k**) patient stories (topic 9); (**l**) symptoms description (topic 12).

The STM model’s unique ability to evaluate textual content using document-level covariates allows us to implement an interaction term of time and sentiment polarity into the STM model. In doing so, we are able to track the evolution of the public sentiment towards each topic, which is subject to change over time. Figure 5 shows the variations in the topic prevalence based on sentiment polarity from August 2021 to July 2022. The x-axis represents time. The y-axis represents the topic proportion. The blue and red lines represent the proportions of negative and positive Weibo posts, and the dashed line represents the 95% confidence interval.

Figure 5a–g shows the evolution of positive topics in terms of sentiment polarity over time. Except for community effect (topic 5), which decreased from 3.6% to 2.8%, none of the positive topics showed a downward trend in the prevalence in positive Weibo posts during the study period. Among them, celebrity effect (topic 4) experienced the greatest increase from 1.2% to 5.6%. Encouragement (topic 10) also reported a noticeable surge from 5.5% to 8.2%. The growths in positive topic proportions of the other four topics are around 1%.

Figure 5h–l represents the changes in negative topics with regard to sentiment polarity during the study period. In particular, public stigma (topic 6) showed a startling increase of 8.7% in the prevalence in negative Weibo posts (1.1–9.8%). Similarly, patient stories (topic 9) reported a two-fold increase in the prevalence in negative Weibo posts (3.6–7.2%) during the study period. By comparison, the upward trend of symptoms description (topic 12) in the prevalence in negative Weibo posts was relatively moderate (1.9–3.5%). On the other hand, mental health service (topic 8) and self-stigma (topic 7) decreased in their respective negative topic proportions by 0.6% (1.7–1.1%) and 0.1% (2.8–2.7%).

## 5. Discussion

Based on the themes, we classified the 12 topics into four factors associated with mental health attitude in China: stigma (*n* = 54,559, 37.21%), mental health literacy (*n* = 32,199, 21.96%), public promotion (*n* = 30,747, 20.97%), and social support (*n* = 29,120, 19.86%). In particular, stigma contains public stigma (topic 6), self-stigma (topic 7), and patient stories (topic 9). Mental health literacy contains information seeking (topic 1), advice and sharing (topic 2), and symptoms description (topic 12). Public promotion includes media promotion (topic 3), celebrity effect (topic 4), and World Mental Health Day (topic 11). Social support includes community effect (topic 5), mental health service (topic 8), and encouragement (topic 10).

Stigma is the primary factor that induces negative mental health attitudes in China. None of the topics related to this factor were considered positive. Public stigma (topic 6) showed a startling increase in negative Weibo posts from 1.1% to 9.8%. Patient stories (topic 9) also reported a two-fold increase in negative Weibo posts during the study period (3.6–7.2%). The existence of stigma in mental health has been explored and confirmed by a large body of literature in psychology and psychiatry [47]. Stigma is a multidimensional construct consisting of four subtypes: public stigma, personal stigma, perceived public stigma, and self-stigma [12]. We detected public stigma (topic 6) and self-stigma (topic 7) in this study. Public stigma refers to prejudice and discrimination against people with mental health problems by the general public [48]. Self-stigma means that mental health patients internalize the public stigma and devalue their self-esteem [49]. As the outbreak and rapid spread of the COVID-19 pandemic triggering enormous mental health problems around the globe [50], the stigma against people with mental health problems is on the rise as well. Stakeholders in the mental health field should strive to reduce the stigma of any kind against people with mental health problems for the continuous improvement of mental health attitude in China.

Mental health literacy refers to knowledge and beliefs about the aspects of mental health and treatment, including causes of illness, types of treatment, and the recovery process [51]. Three topics were classified into this factor. Among them, information seeking (topic 1) and advice and sharing (topic 2) were both identified as positive, and the positivity had been growing during the period of this study, indicating that mental health literacy is very helpful in the improvement of mental health attitude in China. Although symptoms description (topic 12) was identified as a negative topic, its topic prevalence in negative Weibo posts is a mere 1.7%, which is the lowest among the negative topics. This result echoed previous findings that mental health literacy is positively related to mental health attitude [9], which provides an important inspiration for mental health stakeholders that the improvement of mental health literacy could set the groundwork for enhancing mental health attitude in China.

Public promotion is another factor associated with fostering positive mental health attitudes in China. The topics related to this factor were all identified as positive. The public promotion of public health issues, especially from entertainment figures and works, often raises the general public’s interest by making the underlying scientific message temporally more interesting. For example, daily test rates for the breast cancer gene (BRCA) increased by 64% in the 15 business days right after Angelina Jolie’s *New York Times* column detailing her decision to undergo a double mastectomy to prevent breast cancer [52]. The blockbuster film The Day After Tomorrow prompted heavy traffic of global warming websites from 10 days before the release date to 19 days after the release date [53]. In addition, the temporary interest could potentially be transformed into stable interest and foster a tendency to reengage with the message over time [54]. Therefore, to efficiently enhance mental health attitude in China, mental health stakeholders could try to encourage more celebrities to promote mental health and more filmmakers to produce mental health-related work.

Social support indicates a behavioral pattern among people with mental health problems that they prefer to seek help from families, friends, and informal mental health services rather than professionals [55]. It is a critical factor associated with mental health attitude, as it reflects the degree to which society understands and cares about people with mental health problems. The available studies report inconsistent findings about the role of social support in mental health [56]. Some studies report a negative relationship between social support and mental health, while other studies demonstrate opposite findings [57]. This study regarded social support as a positive factor in facilitating mental health attitudes, which is consequently beneficial to mental health. Specifically, community effect (topic 5) and encouragement (topic 10) remained positive throughout the study period. Although mental health service (topic 8) was identified as negative, it was a reflection of the dissatisfaction with the shortage of mental health resources based on the Weibo posts linked to this topic. Therefore, advocating for stronger social support could be considered by stakeholders in the mental health field as a worthy approach to improving mental health attitude in China.

## 6. Conclusions

The mental health field puts significant effort into finding ways to improve mental health and reduce the social and individual problems caused by negative mental health attitudes. This study adds support to previous literature on the identification of the factors associated with mental health attitude in China. We used Weibo, China’s leading social media site, as the data source. The data were processed via the STM model to not only look into the themes of the topics, but also track the evolution of the public sentiment towards the topics over time. With an in-depth analysis of 146,625 Weibo posts, this study captures 12 topics that are, in turn, classified into four factors, namely, stigma (*n* = 54,559, 37.21%), mental health literacy (*n* = 32,199, 21.96%), public promotion (*n* = 30,747, 20.97%), and social support (*n* = 29,120, 19.86%). The results show that stigma is the primary factor inducing negative mental health attitudes in China, as none of the topics related to this factor are considered positive. Mental health literacy, public promotion, and social support are the factors that could enhance positive attitudes towards mental health, since most of the topics related to these factors are identified as positive ones. The findings provide valuable insights for stakeholders in the mental health field to improve mental health attitude in China.

This study has several positive implications for mental health research. First, the use of social media data generates an unprecedented amount of data that is hard to achieve through traditional research methods. The real-time user-generated data enables re-searchers in this field to track the dynamic changes in the mental health of the general public. Second, the adoption of deep learning models to extract and analyze the emotional tendencies expressed in the Weibo posts helps researchers deepen their understanding of the data. Further, the utilization of the structural modeling approach allows researchers to use a variety of covariates to examine the data, which could unlock information supporting new insights in the mental health field.

Every empirical study has limitations. First, although Weibo is arguably the most popular social media site in China, many individuals also express their attitudes towards mental health within other online forums such as Zhihu and WeChat groups. Second, this study only analyzed Weibo texts, while graphic content in Weibo posts also contains important information. Third, posts with extreme sentiments were often removed from social media platforms, which could result in the reduction of the overall percentage of a particular emotion. Last, this study failed to consider different age group’s attitudes to mental health, since Weibo does not contain information showing the age of the posters.

## Figures and Tables

**Table 1:** Topics generated by STM.

Number	Labels	Proportion	Top Words
1	Information seeking	6.66%	mental health, learn, medicine, hospital, Baidu
2	Advice and sharing	8.36%	spread, share, WeChat, private massage, get
3	Media promotion	8.02%	television, education, promotion, postpartum depression, effective
4	Celebrity effect	10.78%	Yang Zi, celebrity, endorse, awareness, attention
5	Community effect	6.39%	prevalence, globe, normal, group, help
6	Public stigma	16.73%	career, pain, housing, fear, anguish
7	Self-stigma	7.28%	doubt, fault, disappoint, own, loneliness
8	Mental health service	6.77%	support, donation, family, work, community
9	Patient stories	13.20%	lockdown, quarantine, Shanghai, discrimination, job
10	Encouragement	6.70%	fighting, strong, faith, warrior, amazing
11	World Mental Health Day	2.17%	Mental Health Day, today, involvement, success, campaign
12	Symptoms description	6.94%	physical, anxiety, depression, anger, sad

**Table 2:** Most representative Weibo texts and topic label selection.

**Information seeking (topic 1):** “Which hospital has the best mental health treatment?”; “Which doctor is best at dealing with postpartum depression?”; “Baidu is full of advertisements. Where can I find some useful information?”
**Advice and sharing (topic 2):** “Help me spread the awareness for mental health!”; “There’s this WeChat group I’m in that people share mental health information everyday”; “You can get useful tips for coping with mental health problems at this website.”
**Media promotion (topic 3):** “Television is so effective in promoting mental health awareness.”; “Female Psychologist is really good mental health education.”; “Whoever in charge of mental health should pay attention to the advantage of media promotion.”
**Celebrity effect (topic 4):** “So many celebrities are advocating mental health right now.”; “I’m a huge fan of Yang Zi. I will followed her lead to help those with mental health problems.”; “Yang Zi is the reason why I pay attention to mental health.”
**Community effect (topic 5):** “Mental health problems are so prevalent. We are not alone.”; “Mental health affects people of all nationalities, races, ethnicities, genders and ages.”; “Thousands of Millions of people have mental health problems globally.”
**Public stigma (topic 6):** “I might lose my job if my boss knows I have mental health problems.”; “They think I’m some kind of a lunatic.”; “My career will be over if anyone knows I am taking mental health treatment.”
**Self-stigma (topic 7):** “I’m so vulnerable. I let myself stuck in this position. I’m feeling depressed lately. But I don’t need to seek help. That’s what cowards do.”; “Why they are perfectly fine with the pressure? Am I the only one feeling mentally unwell?”
**Mental health service (topic 8):** “China has more than 16 million mental health patients. It is imperative to improve mental health services”; “I’m so glad that there are so many mental health resources in my disposal.”; “The government needs to up its game in providing social support to people.”
**Patient stories (topic 9):** “Last two years is the most difficult time of my life.”; “This is such a tragedy. We must prevent it from happening again to someone else.”; “I moved seven times last year to run away from the discrimination. ”
**Encouragement (topic 10):** “Keep fighting! You are not alone!”; “You guys are the true warriors.”; “You guys are amazing! Keep the faith.”
**World Mental Health Day (topic 11):** “Let’s join together to fight mental health problems! #WorldMentalHealthDay.”; “To all those living with mental health problems around the world, keep fighting! #WorldMentalHealthDay”; “We encourage ALL our policymakers to promote World Mental Health Day.”
**Symptoms description (topic 12):** “You should seek mental health care once you have the following symptoms.”; “I didn’t know there would be physical symptoms. Nauseous all the time…”; “Physical symptoms is the hardest part of mental health problems.”

## Data Availability

Data are available by emailing caryfenggan@163.com.
